# Severe Warm Autoimmune Hemolytic Anemia With Profound Anemia Requiring Multimodal Immunosuppressive Therapy

**DOI:** 10.7759/cureus.113303

**Published:** 2026-07-24

**Authors:** Helena Awada, Rohan Chawla, Yanicka Shepherd, Cameron Bondy, David Yetenikyan

**Affiliations:** 1 Internal Medicine, Arrowhead Regional Medical Center, Colton, USA; 2 Family Medicine, Arrowhead Regional Medical Center, Colton, USA

**Keywords:** autoimmune cytopenia, autoimmune hemolytic anemia, autoimmune overlap syndrome, intravenous immunoglobulin, pulse-dose corticosteroids, severe anemia, sjögren syndrome, splenomegaly, systemic lupus erythematosus, warm autoimmune hemolytic anemia

## Abstract

Warm autoimmune hemolytic anemia (wAIHA) is an uncommon but potentially life-threatening autoimmune disorder characterized by immunoglobulin G (IgG)-mediated destruction of erythrocytes. Secondary wAIHA frequently occurs in association with systemic autoimmune diseases, particularly systemic lupus erythematosus (SLE), while Sjögren syndrome represents a less common but recognized association. Fulminant presentations with profound anemia remain rare and require prompt diagnosis, aggressive immunosuppressive therapy, and multidisciplinary management.

We present the case of a 25-year-old woman with a history of reported Sjögren syndrome, Hashimoto thyroiditis, and clinical and serologic features concerning for evolving SLE who developed rapidly progressive direct antiglobulin test (DAT)-positive wAIHA. Despite receiving five units of packed red blood cells (PRBCs) at an outside hospital, her hemoglobin continued to decline after transfer, reaching a nadir of 3.2 g/dL. Laboratory evaluation demonstrated marked hemolysis with reticulocytosis, elevated lactate dehydrogenase, indirect hyperbilirubinemia, undetectable haptoglobin, spherocytes on peripheral smear, and a DAT positive for both IgG and complement (C3). The hospital course was further complicated by thrombocytopenia and severe splenomegaly, raising concern for autoimmune overlap syndrome and possible Evans syndrome. She was successfully treated with pulse-dose intravenous methylprednisolone, intravenous immunoglobulin (IVIG), folic acid supplementation, and transfusion support. Rheumatologic evaluation revealed positive antinuclear antibody (ANA), anti-SSA, antiphospholipid, and anti-thyroid peroxidase antibodies, prompting initiation of hydroxychloroquine. Hemolysis resolved rapidly with normalization of bilirubin, recovery of platelet count, and sustained improvement in hemoglobin, allowing transition to an oral prednisone taper without requiring rituximab or splenectomy.

This case highlights that wAIHA may represent the initial manifestation of evolving systemic autoimmune disease and should be considered in young patients presenting with profound hemolytic anemia. Early recognition, aggressive first-line immunosuppressive therapy, timely transfusion support, and multidisciplinary collaboration can be lifesaving and may achieve complete hematologic recovery without the need for second-line biologic therapy.

## Introduction

Warm autoimmune hemolytic anemia (wAIHA) is the most common subtype of autoimmune hemolytic anemia and is characterized by immunoglobulin G (IgG)-mediated destruction of erythrocytes at body temperature, resulting primarily in Fc receptor-mediated extravascular hemolysis within the spleen [1]. Although uncommon, with an estimated annual incidence of one to three cases per 100,000 individuals, wAIHA remains an important cause of acquired hemolytic anemia because of its potential for rapid clinical deterioration and significant morbidity and mortality [2,3]. Clinical presentation ranges from compensated chronic hemolysis to fulminant anemia associated with hemodynamic instability, tissue hypoxia, and multiorgan dysfunction requiring urgent transfusion and immunosuppressive therapy [1-3].

Approximately one-half of all cases of wAIHA are secondary to an underlying disorder, including lymphoproliferative malignancies, autoimmune diseases, infections, medications, immunodeficiency syndromes, and, less commonly, solid organ malignancies [1,3]. Among autoimmune diseases, systemic lupus erythematosus (SLE) represents one of the most well-recognized causes of secondary wAIHA, whereas Sjögren syndrome is a considerably less common but increasingly recognized association [4-6]. Hematologic abnormalities are common manifestations of systemic autoimmunity and include autoimmune hemolytic anemia, immune thrombocytopenia, leukopenia, neutropenia, and pancytopenia. Importantly, these cytopenias may precede the development of classic rheumatologic manifestations, making autoimmune hemolytic anemia the presenting feature of an evolving systemic autoimmune disorder [4,6].

Patients with overlapping autoimmune diseases, including SLE, Sjögren syndrome, and autoimmune thyroid disease, demonstrate profound immune dysregulation characterized by chronic B-cell activation, autoreactive T-cell responses, complement activation, and production of multiple pathogenic autoantibodies [5,6]. This phenomenon, commonly referred to as polyautoimmunity, increases susceptibility to immune-mediated cytopenias while often complicating diagnosis and management. Distinguishing isolated autoimmune hemolytic anemia from autoimmune overlap syndromes or disorders such as Evans syndrome may therefore be challenging, particularly in patients presenting with thrombocytopenia or multiple positive autoimmune serologies.

The diagnosis of wAIHA relies on both clinical suspicion and laboratory evidence of hemolysis. Typical findings include elevated lactate dehydrogenase, indirect hyperbilirubinemia, reticulocytosis, decreased or undetectable haptoglobin, and spherocytes on peripheral blood smear. Confirmation is obtained through a positive direct antiglobulin test (DAT) demonstrating IgG with or without complement (C3) coating erythrocytes [1]. Patients with complement-positive wAIHA may experience more severe hemolysis because both splenic macrophage-mediated erythrophagocytosis and complement activation contribute to erythrocyte destruction. Severe disease frequently complicates blood bank compatibility testing because warm autoantibodies react broadly with donor erythrocytes, making identification of completely compatible blood difficult. Nevertheless, transfusion should not be delayed in patients with life-threatening anemia, as timely restoration of oxygen-carrying capacity outweighs concerns regarding serologic incompatibility [1,7].

Corticosteroids remain the cornerstone of first-line treatment and achieve initial response rates approaching 70-85% by reducing macrophage-mediated erythrophagocytosis and suppressing autoantibody production [1]. Patients with fulminant hemolysis frequently require escalation of therapy with pulse-dose intravenous corticosteroids, intravenous immunoglobulin (IVIG), and judicious transfusion support guided by clinical status while definitive immunosuppressive therapy takes effect. Rituximab has become the preferred second-line treatment for steroid-refractory or relapsed disease, demonstrating durable remission rates and reducing the need for splenectomy [2,8]. Because management often requires simultaneous treatment of hemolysis, transfusion support, and the underlying autoimmune disease, multidisciplinary collaboration among hematology, rheumatology, transfusion medicine, and internal medicine is essential for optimizing outcomes.

Profound anemia resulting from wAIHA remains uncommon, and reports describing hemoglobin concentrations approaching 3 g/dL are limited. Such presentations represent true hematologic emergencies requiring immediate recognition and coordinated multidisciplinary intervention. Furthermore, cases occurring in the setting of autoimmune overlap syndromes involving reported Sjögren syndrome with evolving SLE remain rare and provide valuable insight into the complex relationship between systemic autoimmunity and immune-mediated cytopenias.

We present the case of a young woman with reported Sjögren syndrome and clinical and serologic features concerning evolving SLE who developed fulminant wAIHA with a hemoglobin nadir of 3.2 g/dL despite multiple packed red blood cell (PRBC) transfusions. Prompt treatment with pulse-dose intravenous methylprednisolone, IVIG, and supportive transfusion therapy resulted in complete hematologic recovery without the need for rituximab or splenectomy. This case highlights the importance of recognizing severe autoimmune hemolytic anemia as a potentially life-threatening manifestation of systemic autoimmunity, emphasizes the diagnostic challenges posed by autoimmune overlap syndromes, and demonstrates that timely, aggressive first-line immunosuppressive therapy can achieve excellent outcomes even in patients presenting with profound anemia.

## Case presentation

A 25-year-old woman with a past medical history of reported Sjögren syndrome, Hashimoto thyroiditis status post hemithyroidectomy, chronic migraines, and depression was transferred to our tertiary care center for evaluation and management of severe autoimmune hemolytic anemia after requiring multiple blood transfusions at an outside hospital.

Five days prior to her initial presentation, she developed a severe left-sided migraine associated with nausea, vomiting, and photophobia. She denied fever, recent illness, medication changes, travel, toxic exposures, recent infections, significant cold exposure (including occupational or environmental exposure), or cold-induced symptoms. Evaluation at the outside hospital revealed profound anemia with a hemoglobin concentration of 5.6 g/dL and persistent sinus tachycardia. She received two units of PRBCs; however, her hemoglobin demonstrated only transient improvement and continued to decline despite transfusion support. During her hospitalization, she ultimately required a total of five units of PRBCs. Initial laboratory evaluation demonstrated a positive DAT, elevated total bilirubin (4.6 mg/dL), reticulocytosis (15%), and folate deficiency, raising concern for autoimmune hemolytic anemia. Iron studies demonstrated a serum iron concentration of 184 µg/dL, total iron-binding capacity of 273 µg/dL, transferrin saturation of 67%, and a ferritin concentration of 1,739 ng/mL. These findings were not consistent with iron deficiency, and intravenous iron supplementation was therefore not indicated. She received prednisone 60 mg before transfer to our institution for higher-level hematologic care.

Upon arrival, the patient reported a six-month history of progressively worsening fatigue, exertional dyspnea, palpitations, tinnitus, and generalized weakness. She also described a one-week history of dark-colored urine and jaundice. She denied any prior history of anemia, blood transfusions, thromboembolic disease, or known hematologic disorders. Family history was negative for inherited blood disorders or autoimmune hemolytic anemia.

On examination, she appeared ill, fatigued, and markedly pale with scleral icterus and persistent tachycardia. Cardiovascular examination revealed a regular rhythm without murmurs. Pulmonary examination was unremarkable. Abdominal examination demonstrated mild right upper quadrant tenderness without rebound or guarding. No palpable lymphadenopathy or hepatosplenomegaly was appreciated on physical examination.

Initial laboratory studies demonstrated a hemoglobin concentration of 6.1 g/dL, which rapidly declined despite transfusion support, reaching a nadir of 3.2 g/dL during hospitalization. The platelet count decreased from 140 × 10⁹/L to 70 × 10⁹/L, while the reticulocyte count increased to 31.4%, reflecting an appropriate marrow response to severe hemolysis. Lactate dehydrogenase exceeded 1,000 U/L, total bilirubin peaked at 4.6 mg/dL with a direct bilirubin of 1.2 mg/dL (calculated indirect bilirubin approximately 3.4 mg/dL), and haptoglobin was undetectable. Peripheral blood smear demonstrated spherocytes without schistocytes or other features of microangiopathic hemolysis. The DAT was positive for both IgG and C3, confirming immune-mediated hemolysis. The laboratory report documented DAT positivity for both IgG and C3; however, the specific testing methodology (polyspecific versus monospecific sequence) was not available. Blood bank antibody identification demonstrated an anti-S alloantibody together with warm and cold autoantibodies, and compatible S-negative red blood cell units were recommended for transfusion. Because IgM characterization and thermal amplitude studies were unavailable, a definitive distinction between complement-positive wAIHA and mixed AIHA could not be established. Coagulation studies demonstrated an isolated prolonged activated partial thromboplastin time (50.9 seconds) with normal prothrombin time and international normalized ratio. Serial laboratory trends throughout hospitalization are summarized in Table 1. 

**Table 1:** Serial laboratory trends demonstrating severe hemolysis and hematologic recovery during hospitalization. Laboratory values obtained at presentation, peak disease severity, and discharge are shown. The patient exhibited profound warm autoimmune hemolytic anemia with a hemoglobin nadir of 3.2 g/dL, marked reticulocytosis, elevated lactate dehydrogenase, undetectable haptoglobin, hyperbilirubinemia, thrombocytopenia, and a positive direct antiglobulin test for immunoglobulin G and complement component 3. Following treatment with pulse-dose intravenous methylprednisolone, intravenous immunoglobulin (IVIG), transfusion support, and an oral corticosteroid taper, hemoglobin and platelet counts recovered with normalization of bilirubin. DAT: direct antiglobulin test; LDH: lactate dehydrogenase; IgG: immunoglobulin G; C3: complement component 3; IVIG: intravenous immunoglobulin

Laboratory Parameter	Units	Reference Range	Presentation	Peak/Nadir	Discharge
Hemoglobin	g/dL	12.0–16.0	6.1	3.2	11
Platelet count	×10⁹/L	150–400	140	70	223
Reticulocyte count	%	0.5–2.5	15	31.4	Not Recorded
Lactate dehydrogenase (LDH)	U/L	140–280	>1000	>1000	Improved
Total bilirubin	mg/dL	0.2–1.2	4.6	4.6	0.9
Haptoglobin	mg/dL	30–200	Undetectable	Undetectable	Not Recorded
Direct antiglobulin test (DAT)	—	Negative	IgG+C3 positive		

Because of rapidly progressive hemolysis and critical anemia, the hematology service was consulted immediately. Pulse-dose intravenous methylprednisolone (1 g daily) was initiated along with IVIG (1 g/kg/day) for three consecutive days (hospital days 1-3). Following the transfer, the patient required four additional units of PRBCs. Transfusion was administered to maintain hemoglobin above 7 g/dL while definitive immunosuppressive therapy took effect despite anticipated compatibility challenges. Folic acid supplementation (5 mg daily) was also initiated. Given the severity of the disease, rituximab was considered as second-line therapy should she fail to demonstrate an adequate response to corticosteroids and IVIG.

The concurrent development of thrombocytopenia prompted concern for an underlying systemic autoimmune disorder and possible Evans syndrome. Rheumatology was consulted for further evaluation. Autoimmune testing demonstrated a positive antinuclear antibody (ANA) at 1:640 with a homogeneous nuclear pattern, positive anti-SSA antibodies, positive anticardiolipin antibodies, positive anti-thyroid peroxidase antibodies, and positive antiphosphatidylserine IgG antibodies. Anti-double-stranded DNA antibodies were negative, complement levels (C3 and C4) remained within normal limits, and paroxysmal nocturnal hemoglobinuria (PNH) flow cytometry was negative. The prolonged activated partial thromboplastin time, together with positive anticardiolipin antibodies, raised concern for lupus anticoagulant; however, confirmatory lupus anticoagulant testing was not available. A summary of the autoimmune and hematologic evaluation is provided in Table 2. In conjunction with her reported history of Sjögren syndrome, thrombocytopenia, autoimmune hemolytic anemia, and a faint malar rash observed during hospitalization, these findings raised concern for evolving SLE. Hydroxychloroquine 200 mg daily was initiated.

**Table 2:** Autoimmune and hematologic evaluation supporting secondary warm autoimmune hemolytic anemia. Autoimmune and hematologic studies obtained during hospitalization demonstrated an ANA 1:640, positive anti-SSA antibodies, positive anticardiolipin antibodies, positive antiphosphatidylserine IgG, and positive anti-thyroid peroxidase antibodies, consistent with an underlying autoimmune diathesis. The DAT was positive for both immunoglobulin G (IgG) and complement component 3 (C3), confirming warm autoimmune hemolytic anemia with complement involvement. Blood bank antibody identification demonstrated an anti-S alloantibody in addition to warm and cold autoantibodies, and compatible S-negative red blood cell units were recommended for transfusion. Because IgM characterization and thermal amplitude studies were unavailable, a definitive distinction between complement-positive warm AIHA and mixed AIHA could not be established. The laboratory report documented DAT positivity for both IgG and C3; however, the specific testing methodology (polyspecific versus monospecific sequence) was not available. PNH flow cytometry was negative, excluding PNH as an alternative etiology. ANA: antinuclear antibody; SSA: anti-Sjögren syndrome-related antigen A; dsDNA: anti-double-stranded DNA; C3: complement component 3; C4: complement component 4; anti-TPO: anti-thyroid peroxidase antibody; PNH: paroxysmal nocturnal hemoglobinuria; DAT: direct antiglobulin test; IgG: immunoglobulin G

Test	Result	Reference/Interpretation
Antinuclear antibody (ANA)	Positive (1:640, homogeneous nuclear pattern)	Negative
Anti-SSA (anti-Sjögren syndrome-related antigen A)	Positive	Negative
Anticardiolipin antibody	Positive	Negative
Antiphosphatidylserine IgG	Positive	Negative
Anti-thyroid peroxidase (Anti-TPO)	Positive	Negative
Anti-double-stranded DNA (dsDNA)	Negative	Negative
Complement component 3 (C3)	Normal	Normal
Complement component 4 (C4)	Normal	Normal
Direct antiglobulin test (DAT)	IgG and C3 positive	Negative
Paroxysmal nocturnal hemoglobinuria (PNH) flow cytometry	Negative	Negative

**Figure 1:**
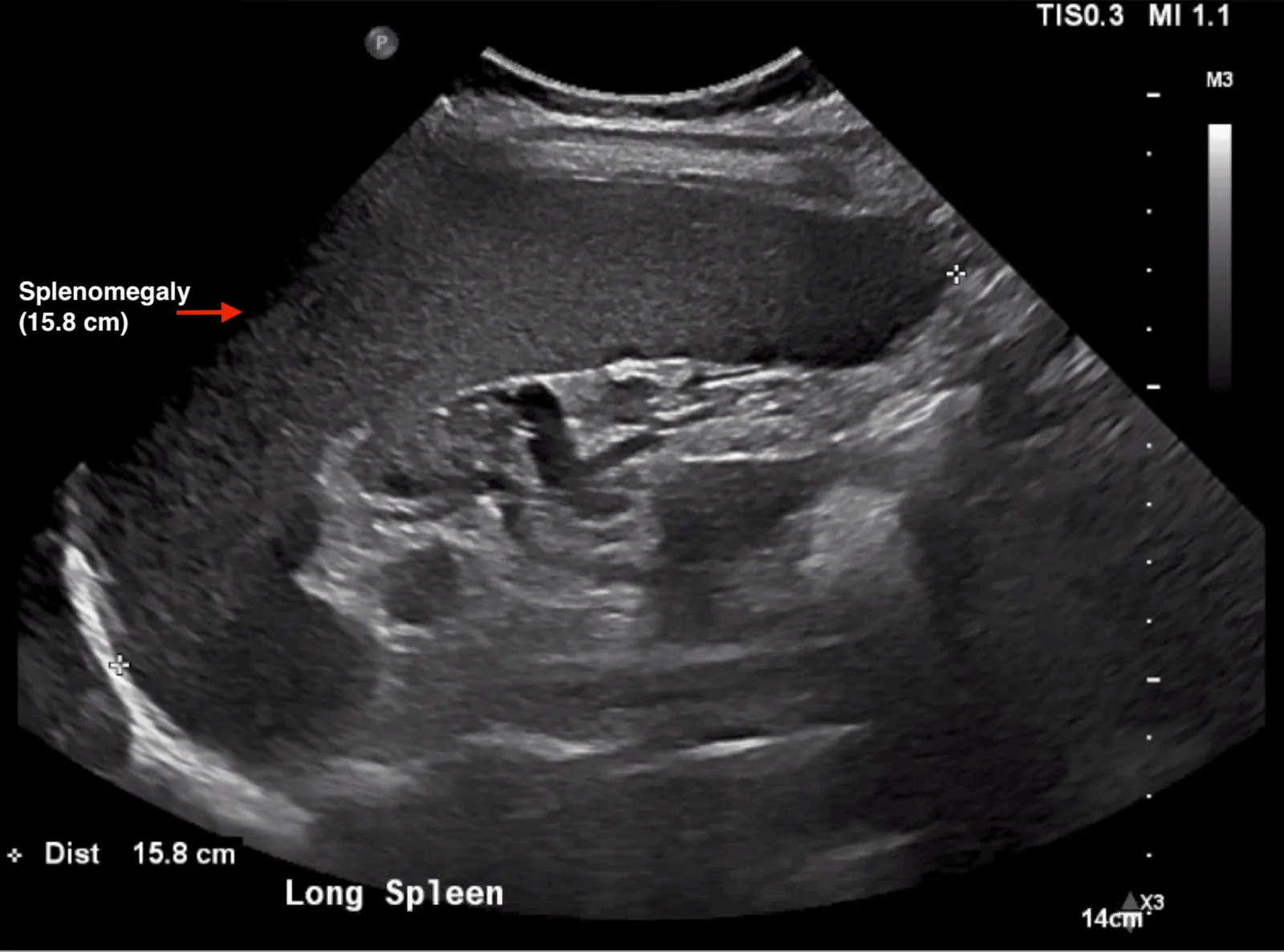
Longitudinal abdominal ultrasonography demonstrating moderate-to-severe splenomegaly. The red arrow indicates the enlarged spleen, which measured 15.8 cm in maximal longitudinal dimension, consistent with increased extravascular hemolysis and splenic sequestration of immunoglobulin G (IgG)-coated erythrocytes in warm autoimmune hemolytic anemia.

During hospitalization, the patient developed diffuse abdominal pain, most pronounced in the right upper quadrant. Abdominal ultrasonography demonstrated moderate-to-severe splenomegaly and diffuse gallbladder wall thickening without cholelithiasis (Figure 1). Subsequent hepatobiliary iminodiacetic acid (HIDA) scanning demonstrated no evidence of acute cholecystitis. She also experienced persistent severe headaches with photophobia following IVIG administration. Computed tomography of the head demonstrated no acute intracranial hemorrhage or other intracranial abnormality, and her symptoms were attributed to IVIG-associated headache.

Given the severity of anemia and associated thrombocytopenia, bone marrow biopsy was considered to exclude an underlying marrow disorder or lymphoproliferative process. However, as the patient's hematologic parameters improved rapidly with immunosuppressive therapy, the procedure was deferred, with plans for outpatient evaluation only if recurrent or persistent cytopenias developed.

Following completion of pulse-dose corticosteroids and IVIG, laboratory improvement paralleled the patient's clinical recovery. Hemoglobin increased from 3.2 g/dL to 6.1 g/dL, then 8.9 g/dL, and ultimately 11.0 g/dL by discharge. Concurrently, platelet count recovered from a nadir of 70 × 10⁹/L to 223 × 10⁹/L, while total bilirubin normalized from 4.6 mg/dL to 0.9 mg/dL without additional transfusion requirements (Table 1), demonstrating rapid resolution of hemolysis. Because of this sustained clinical response, rituximab was ultimately deferred. Intravenous methylprednisolone was transitioned to an oral prednisone taper beginning on hospital day 10, while hydroxychloroquine and folic acid were continued.

By hospital day 13, the patient remained hemodynamically stable with no evidence of ongoing hemolysis or additional transfusion requirements. She reported significant improvement in fatigue, dyspnea, and jaundice, and was discharged home on an oral prednisone taper, hydroxychloroquine, and folic acid with close outpatient follow-up by hematology and rheumatology.

## Discussion

wAIHA accounts for approximately 70-80% of all autoimmune hemolytic anemia cases and is characterized by IgG-mediated erythrocyte destruction occurring primarily through Fc receptor-mediated clearance by splenic macrophages [1,2]. While approximately one-half of cases are idiopathic, the remainder occur secondary to underlying conditions, including lymphoproliferative disorders, medications, infections, and systemic autoimmune diseases [1,3]. Among autoimmune disorders, SLE is one of the best-recognized causes of secondary wAIHA, whereas Sjögren syndrome represents a less common but increasingly recognized association [4-6].

Our patient presented with fulminant wAIHA in the setting of reported Sjögren's syndrome and multiple serologic and clinical findings concerning evolving SLE. Although she carried a prior diagnosis of Sjögren syndrome, hospitalization revealed a high-titer ANA (1:640), positive anti-SSA antibodies, antiphospholipid antibodies, thrombocytopenia, autoimmune hemolytic anemia, and a faint malar rash, prompting concern for an evolving lupus phenotype despite negative anti-double-stranded DNA antibodies and preserved complement levels. This case illustrates an important clinical principle: autoimmune cytopenias may precede fulfillment of formal SLE classification criteria and may represent one of the earliest manifestations of systemic autoimmunity [4,9]. Consequently, patients presenting with immune-mediated cytopenias should undergo a comprehensive rheumatologic evaluation, particularly when multiple autoimmune features coexist.

The coexistence of reported Sjögren syndrome, Hashimoto thyroiditis, and autoimmune hemolytic anemia in this patient also highlights the concept of polyautoimmunity, in which multiple autoimmune diseases coexist because of shared genetic susceptibility and common immunologic pathways. Chronic B-cell activation, impaired immune tolerance, autoreactive T-cell signaling, and production of pathogenic autoantibodies contribute to immune-mediated tissue injury across multiple organ systems [5,6]. Sjögren syndrome has traditionally been regarded as an exocrine gland disorder; however, it is increasingly recognized as a systemic autoimmune disease capable of producing hematologic manifestations including leukopenia, thrombocytopenia, hypergammaglobulinemia, lymphoma, and, less commonly, autoimmune hemolytic anemia [5]. Recognition of these overlapping autoimmune conditions is clinically important because hematologic abnormalities may be the initial or dominant manifestation of systemic disease.

The diagnosis of wAIHA requires demonstration of both hemolysis and immune-mediated erythrocyte destruction. Our patient exhibited nearly every characteristic diagnostic feature of the disease, including profound anemia, marked reticulocytosis, elevated lactate dehydrogenase, indirect hyperbilirubinemia, undetectable haptoglobin, spherocytes on peripheral blood smear, and a DAT positive for both IgG and complement (C3). Diagnostic findings supporting wAIHA are summarized in Table 3, while serial laboratory recovery following therapy is demonstrated in Table 1. Although most patients with wAIHA demonstrate IgG positivity alone, approximately 20-30% also exhibit complement deposition [1,2]. The updated 2024 classification of autoimmune hemolytic anemia emphasizes that positivity for both IgG and C3 on the DAT may occur in either classic wAIHA or mixed AIHA [10]. In this patient, blood bank antibody identification detected both warm and cold autoantibodies in addition to an anti-S alloantibody. However, IgM characterization and thermal amplitude studies were not available, precluding definitive classification as mixed AIHA. Nevertheless, the patient's predominant clinical presentation, absence of clinically significant cold-induced symptoms, and excellent response to corticosteroids and IVIG supported treatment as wAIHA with complement activation [10]. Complement-positive disease may be associated with more severe hemolysis because erythrocyte destruction occurs through both splenic macrophage-mediated clearance and complement activation, likely contributing to the profound hemolysis observed in this patient.

**Table 3:** Clinical and laboratory findings consistent with warm autoimmune hemolytic anemia. Summary of the patient's diagnostic findings demonstrating active immune-mediated hemolysis. The combination of severe anemia (hemoglobin nadir 3.2 g/dL; reference range: 12.0–16.0 g/dL), reticulocytosis (31.4%; reference range: 0.5–2.5%), elevated lactate dehydrogenase (LDH >1,000 U/L; reference range: 140–280 U/L), hyperbilirubinemia (total bilirubin 4.6 mg/dL; direct bilirubin 1.2 mg/dL), undetectable haptoglobin (reference range: 30–200 mg/dL), a positive direct antiglobulin test (DAT) for immunoglobulin G (IgG) and complement component 3 (C3), and spherocytes on peripheral blood smear established the diagnosis of warm autoimmune hemolytic anemia (wAIHA). The absence of schistocytes made microangiopathic hemolytic anemia unlikely, while the favorable response to corticosteroids and IVIG further supported the diagnosis. LDH: lactate dehydrogenase; DAT: direct antiglobulin test; IgG: immunoglobulin G; C3: complement component 3; IVIG: intravenous immunoglobulin

Diagnostic Finding	Patient Result	Interpretation
Hemoglobin	3.2 g/dL nadir	Severe anemia
Reticulocytosis	Present	Supports hemolysis
LDH	Elevated	Supports hemolysis
Hyperbilirubinemia	Elevated (total bilirubin 4.g mg/dL; direct bilirubin 1.2 mg/dL)	Supports hemolysis
Haptoglobin	Undetectable	Supports hemolysis
DAT	IgG and C3 positive	Confirms immune-mediated hemolysis
Peripheral smear	Spherocytes present; schistocytes absent	Consistent with wAIHA
Response to corticosteroids and IVIG	Rapid clinical improvement	Supports diagnosis

One of the most remarkable aspects of this case was the severity of anemia. Despite transfusion of multiple PRBC units, the patient's hemoglobin continued to decline, reaching a nadir of 3.2 g/dL. Hemoglobin concentrations approaching 3 g/dL are rarely reported in adults with wAIHA and generally appear only in isolated case reports. At these levels, oxygen delivery becomes critically compromised, placing patients at risk for myocardial ischemia, high-output heart failure, cerebral hypoxia, arrhythmias, and death [7]. The favorable outcome observed in this case underscores the importance of early recognition and immediate initiation of immunosuppressive therapy before irreversible end-organ injury develops.

Management of severe wAIHA presents significant transfusion challenges because broadly reactive warm autoantibodies frequently interfere with serologic compatibility testing. Historically, concerns regarding transfusion-related hemolysis resulted in delays in blood product administration. Current evidence, however, emphasizes that transfusion should not be withheld in patients with life-threatening anemia solely because completely compatible blood cannot be identified [1,7]. Instead, collaboration with transfusion medicine specialists to provide the safest available or least-incompatible units is recommended while definitive immunosuppressive therapy is initiated. In our patient, transfusion was administered because of profound symptomatic anemia with ongoing hemolysis despite compatibility challenges. Following the transfer, the patient received four additional units of PRBCs to maintain a hemoglobin concentration above 7 g/dL while immunosuppressive therapy took effect. This approach is consistent with current recommendations that transfusion should not be delayed in patients with life-threatening anemia, even when completely compatible blood cannot be identified.

Current international guidelines recommend corticosteroids as first-line therapy for wAIHA, with initial response rates approaching 70-85% [1,2]. Corticosteroids decrease macrophage-mediated erythrophagocytosis and suppress autoantibody production, making them the cornerstone of treatment. Because our patient demonstrated rapidly progressive hemolysis with a critically low hemoglobin concentration, pulse-dose intravenous methylprednisolone (1 g daily) was selected rather than conventional oral corticosteroids. Although prospective data evaluating pulse-dose therapy remain limited, this strategy is widely employed in fulminant autoimmune disease because of its rapid immunosuppressive effects and extensive clinical experience in severe immune-mediated disorders.

IVIG was administered concurrently as adjunctive therapy. Unlike immune thrombocytopenia, IVIG has a less predictable role in wAIHA and is generally reserved for severe or refractory disease [11]. Proposed mechanisms include Fc receptor blockade, modulation of macrophage activity, and interference with pathogenic autoantibody function. Although responses are often transient, IVIG may provide sufficient disease control while corticosteroids achieve maximal therapeutic effect. In our patient, pulse-dose corticosteroids combined with IVIG resulted in rapid resolution of hemolysis, normalization of bilirubin, cessation of transfusion requirements, and sustained hematologic recovery.

Other rescue therapies have been described for refractory or fulminant AIHA. Therapeutic plasma exchange may provide temporary control of severe hemolysis while immunosuppressive therapy becomes effective, although supporting evidence remains limited. Complement-directed therapy with eculizumab has also been reported in selected cases with severe complement-mediated hemolysis, but it is not routinely recommended for classic wAIHA. Because our patient demonstrated rapid improvement following corticosteroids, IVIG, and transfusion support, neither therapeutic plasma exchange nor complement inhibition was required.

An important therapeutic consideration was whether escalation to rituximab would be necessary. Increasing evidence supports earlier incorporation of rituximab in patients with severe wAIHA because combination therapy with corticosteroids improves complete remission rates and reduces relapse compared with corticosteroids alone [2,8]. Nevertheless, rituximab is associated with prolonged B-cell depletion, hypogammaglobulinemia, infusion reactions, and infectious complications. Because our patient demonstrated prompt normalization of bilirubin, stabilization of hemoglobin, recovery of platelet count, and elimination of transfusion requirements following first-line therapy, escalation to rituximab was deferred. This case demonstrates that even patients presenting with profound anemia may achieve durable remission with aggressive first-line immunosuppressive therapy when treatment is initiated promptly.

The concurrent thrombocytopenia represented another important diagnostic challenge. The coexistence of autoimmune hemolytic anemia and thrombocytopenia raised concern for Evans syndrome, a rare autoimmune disorder characterized by simultaneous or sequential immune-mediated destruction of erythrocytes and platelets [12]. However, thrombocytopenia is also a well-recognized hematologic manifestation of systemic autoimmune diseases, particularly SLE. Because immune thrombocytopenia was never definitively established and platelet counts recovered rapidly with treatment directed at hemolysis, Evans syndrome remained a diagnostic consideration rather than a confirmed diagnosis. This distinction has important prognostic implications, as Evans syndrome generally follows a chronic relapsing course requiring prolonged immunosuppressive therapy.

Another notable feature of this case was the presence of moderate-to-severe splenomegaly demonstrated on abdominal ultrasonography (Figure 1). Although splenomegaly is not universally present in patients with wAIHA, when present, it reflects increased splenic macrophage activity and extravascular sequestration of IgG-coated erythrocytes and supports ongoing extravascular hemolysis. The enlarged spleen observed in our patient raises suspicion for active immune-mediated erythrocyte destruction and likely reflects the extraordinary burden of ongoing hemolysis that contributed to her profound anemia.

Bone marrow biopsy was initially considered because of profound anemia and thrombocytopenia. However, progressive normalization of both hemoglobin and platelet count following immunosuppressive therapy strongly supported peripheral immune-mediated destruction rather than intrinsic bone marrow pathology. Current consensus recommendations reserve bone marrow examination for patients with persistent unexplained cytopenias, atypical laboratory findings, suspected lymphoproliferative disease, or inadequate response to therapy [1]. Accordingly, the biopsy was deferred, with plans for outpatient evaluation only if recurrent cytopenias developed.

Although cases describing severe wAIHA associated with Sjögren syndrome have been reported, presentations involving a hemoglobin nadir of 3.2 g/dL remain exceedingly uncommon. Our case demonstrates that aggressive first-line therapy with pulse-dose corticosteroids and IVIG can successfully reverse fulminant immune-mediated hemolysis and avoid escalation to biologic therapy in carefully selected patients. Continued longitudinal follow-up will be important to monitor for recurrent cytopenias and further evolution of systemic autoimmune disease.

## Conclusions

wAIHA is an uncommon but potentially life-threatening condition requiring prompt recognition and timely immunosuppressive therapy. This case demonstrates successful management of fulminant secondary wAIHA associated with evolving systemic autoimmunity using corticosteroids, IVIG, and carefully guided transfusion support without the need for rituximab or splenectomy. Although the patient's DAT demonstrated both IgG and C3 positivity, additional testing to definitively exclude mixed AIHA was unavailable and represents a limitation of this report. Early multidisciplinary management, prompt treatment of severe symptomatic anemia, and continued rheumatologic follow-up remain essential to optimize outcomes in patients presenting with autoimmune hemolytic anemia.
